# "The Hospital" Nursing Section

**Published:** 1906-09-29

**Authors:** 


### The Hospital.

### Nursing Section.

Contributions for "THE HOSPITAL," should be addressed to the EDITOR, "THE HOSPITAL" NURSING SECTION, 28 & 29 Southampton Street, Strand, London, W.C.

No. 1,045.—VOL. XL. SATURDAY, SEPTEMBER 29, 1906.

### Notes on News from the Nursing World.

### THE NURSES' HOSTEL BOARD AND MISS HUME.

THE action of the Board of the Nurses' Hostel, Limited, in reference to Miss Hume, of which details are given on another page, is very unusual, and it seems to us high-handed and unjust. Miss Hume, who is well known in the nursing world and has an excellent record, was only appointed lady superintendent of the Nurses' Hostel three weeks ago. She has now been requested to leave at once, and to accept a cheque for a quarter's salary in lieu of notice. The reason given by the Board is that she had failed in tact, and by Miss C. J. Wood, who is, of course, an influential member of the Board, as well as founder of the Hostel, that she was not satisfied with the state of affairs, and thought that there was a lack of discipline and a disposition to stir up strife. These intimations do not form an adequate explanation for the course pursued, and we think that, in justice to Miss Hume, upon whose capacity and character Miss Wood says she casts no reflection, a further statement from the Board should be forthcoming. We confess that we do not quite understand Miss Wood's position. She appears to have acted as the representative of the Board, and at the same time to have assumed full authority in the Hostel since notice was given to Miss Hume, apparently for the purpose of enforcing that strict discipline which, according to her own statement, is necessary in a nurses' hotel. Her accusation of want of tact on the part of Miss Hume is hardly supported by the fact that the latter has gained the favour of a large number of the nurse inmates, and these are not likely to let the matter rest where it is.

### THE MIDWIVES ACT AND THE UNTRAINED MIDWIFE.

WHILE we printed last week with pleasure a letter on the Midwives Act from a correspondent whose opinions are entitled to every consideration, we think it necessary to say that they do not, in our judgment, affect the position. We neither attacked the motives of the promoters of the Act nor found fault with the administration of it. The promoters are entitled to the utmost credit for their disinterested efforts to substitute trained and competent midwives for untrained and incompetent women. The Act under the existing Board has, on the whole, been administered fairly and with a high sense of responsibility, the refusal to recognise certain well-known schools of training for nurses being the most serious mistake, and one which may have tended to substantially limit the supply of qualified midwives. But our correspondent "W," who demurs to our conclusion, confirms our contention that the Act has failed to accomplish the purpose for which it was enacted. We do not affirm that success in the future is impossible, but we do maintain that so far the results have been disappointing. Some of the reasons for this are supplied by "W"; but whatever the reasons, the fact remains. The view that unless, or until fully-trained midwives can earn enough money to enable them to live, mere registration will evidently prove insufficient to keep the untrained ones from practising as they did before the Act was passed, is not in the least inconsistent with the earnest desire that by some means a much more satisfactory condition of affairs may be achieved.

### WELL DONE, GRANTHAM.

THE account given in another column of the nursing of the injured by the accident on the Great Northern Railway last week at Grantham Hospital shows how a small institution, under good management, can supply the needs of a great emergency. Among the many interesting details, we notice with pleasure the care which was taken of the clothes of the patients when they were removed, so that in case of death there should be no difficulty in speedy identification. There is no doubt that the danger of collapse was materially diminished by the prompt measures which were taken under the directions of the matron to get the sufferers to bed as soon as possible; and it must be very satisfactory to the entire staff that they are all doing well.

### NURSES CENSURED BY A JURY.

AT an inquest held by the coroner at Monaghan last week two nurses were severely censured by the jury. It appears that a female patient was admitted to the county asylum in April suffering from acute melancholia. Owing to a great improvement in her condition she was removed from the special observation ward and given some work to do. One morning the medical man was summoned to her, and found her with marks on her neck and discoloration. Although he tried artificial respiration for an hour, she died from strangulation. Several nurses employed at the asylum gave evidence. One of these, Nurse Daly, stated that when she saw the deceased there was a piece of linen round her neck, which had been used as a cord; but she could not say who removed it. Another affirmed that one of the nurses inculpated told her that she saw a string round the patient's neck. This was flatly denied by one of the accused, who swore that she said nothing of the sort and saw no cord of any kind. As a matter of fact, the piece of linen had been torn off the patient's bed, but the nurse who made the bed did not mention it. The coroner, in summing up, observed that he could place no reliance upon the evidence of the two nurses, and warmly complimented Miss Daly on the assistance she had afforded in finding out the cause of death. We imagine that it will not be easy for the censured nurses to obtain fresh positions.

### NIGHT WORK AND OPERATIONS.

The matron of the Royal Isle of Wight County Hospital, in the course of an interview with our Commissioner reported on another page, states that she is in favour of a probationer becoming accustomed to night work fairly soon in her career. This being her opinion, the probationers at the Isle of Wight Hospital begin night nursing at the end of three months if they appear fit to undertake it. The period is shorter than many other matrons consider desirable, but there is no necessity for a hard and fast rule, so long, of course, as there is always an experienced night nurse in charge. The matron herself invariably attends operations at the Isle of Wight Hospital, and in these circumstances we see no objection in small hospitals to a probationer in a second year having charge of the theatre for three months at a time in order to gain valuable experience.

### A FATAL ACT OF CARELESSNESS.

It is fortunately seldom that an act of carelessness on the part of a nurse has had disastrous results as in the case of an inmate of the Union Workhouse at Ormskirk. The man was suffering from an injured hip, and the nurse had received instructions to apply a liniment. She poured the latter into a pot labelled "two tablespoonfuls," and during her temporary absence the patient drank it and died. Another inmate, who gave evidence, said that the deceased inquired if it was for him, and witness replied that, as there was no one in the other bed, it must be. The nurse, in defence, stated that she did not at once rub the leg as the man was having his tea, and that, when she was leaving the ward, she told him not to touch the liniment. The superintendent nurse admitted that it was very unwise to place poison by a patient's bedside, but the coroner to a certain extent exonerated the nurse, and observed that every one was liable to make mistakes. As usual, now that mischief has been done, regulations are to be framed to render the recurrence of such disasters impossible.

### A CURIOUS SITUATION AT AUCKLAND.

At the last meeting of the Auckland Guardians a special report of a Committee appointed to deal with the nursing arrangements was considered. The principal recommendation of the Committee was that the nursing staff should be reduced. There is a practical difficulty, however, for a proposal that the Local Government Board should be asked to send down a medical inspector to meet the Committee in order that they might be informed of the number of nurses actually required, was met by the rejoinder that it was useless to call in an inspector from Whitehall because their own medical officer, who is responsible to the Local Government Board, had already said that the number is necessary. The report was, nevertheless, adopted by nineteen votes to eight. But we do not believe that the Local Government Board will lend any sanction to the policy which the medical officer affirms is inimical to the well-being of the patients in the infirmary.

### A CANDIDATE FOR REGISTRATION UNDER THE MIDWIVES ACT.

At Battersea on Tuesday an inquiry was held concerning the death of a child of three weeks. The father stated that ten days after the infant's birth the mother was sent to an asylum, and he got a married woman, who said that she had previously been a trained nurse and was now seeking registration under the Midwives Act, to look after the child. On her own showing at the inquest this person only fed the child four times a day, and when the latter was taken under the auspices of the National Society for the Prevention of Cruelty to Children to Wandsworth Infirmary, who had intervend at the instance of neighbours, it weighed only 3½ lb., and was too feeble to cry. The jury added a rider to the usual verdict that death was accelerated by neglect. Whatever subsequent proceedings may, or may not, be taken, we presume that the intending midwife will seek registration in vain.

### MEMORIAL TO AN ARMY SISTER.

An interesting ceremony took place in the Hospital Chapel, Royal Herbert Hospital, Woolwich, on Thursday afternoon, when the Chaplain-General, Bishop Taylor-Smith, dedicated a small brass tablet to the memory of the late Sister Margaret Kendall, who died in April last at Wynberg, South Africa. The service was choral, and was attended largely by the relatives and friends of the deceased, the nursing staff, officers and non-commissioned officers of the Royal Army Medical Corps, and various officers of the garrison and their wives. Bishop Taylor-Smith in a few well-chosen words dedicated the tablet, the words on which were as follows: "To the glory of God, and in loving memory of Sister Margaret Kendall, Queen Alexandra's Imperial Military Nursing Service, who died at Wynberg, 7 April, 1906, aged 29 years. 'Blessed are the pure in heart, for they shall see God.' Erected by her fellow-workers at Woolwich."

### NURSES' SOCIAL UNION.

The members of the Taunton Branch of the Nurses' Social Union met, by kind invitation of Mrs. Platt, at the Vicarage, Kingston, on September 14. The assistant secretary of the Union, Miss Platt, gave them a warm welcome and succeeded in making everyone spend a cheerful and friendly afternoon, partly in listening to Miss Curtis's brightly-written paper, and partly in playing games and strolling in the sunshiny garden. Miss Curtis, superintendent of the Hammersmith District Nursing Association, broached a very wide subject, and one that gave her hearers much food for thought. In her paper, which was a happy combination of earnestness and humour, she insisted that it was impossible for nursing to stand alone, cut off from the other organisations which exist in order to help mankind on the upward path. She also emphasised the importance of the present-day movement in favour of "school nursing."

### LECTURES AT HUDDERSFIELD.

A series of lectures is to be delivered under the auspices of the Huddersfield and District Victoria Sick Poor Nurses' Association by Miss Abraham, the superintendent, under the title of "Home Nursing." It is stated that there was a request for the lectures, and that they will embrace "all matters pertaining to the nursing of sick cases in the homes of the people." We do not doubt the capacity of Miss Abraham—who is not only a fully qualified nurse, but was a successful lecturer under the London School Board—to impart sound instructions; but here again, as in the case of the Axminster classes, there is a danger of persons attending the lectures acquiring, as they consider, sufficient knowledge to try their hand at nursing their friends without consulting a medical man. The risks of pursuing this course in the event of any but the most simple illness should always be emphasised.

### DISPENSING WITH THE NIGHT NURSE.

Notwithstanding that the Frome Guardians have confessed the failure of their experiment to limit night nursing in the Workhouse Infirmary to a midnight visit of the assistant nurse, the Weymouth Guardians have just determined to embark on a similar enterprise. It is due to the latter to say that they do not consider one visit sufficient. The superintendent nurse is to go into the wards between eleven and twelve, and two other nurses are to undertake the night duty or early duty alternate weeks, the former being required to go round all the wards at 3 a.m. Her duty is then to cease, and between 6 and 7 a.m. the nurse taking early duty is to visit all the wards, and afterwards be available for her ordinary work during the day. This elaborate scheme for rendering the appointment of a special night nurse unnecessary may save a little money; but supposing a patient urgently requires attention between the hours of 3 and 6 a.m. what will happen then?

### VACANCIES IN QUEEN ALEXANDRA'S MILITARY NURSING SERVICE.

We are officially informed that there are a few vacancies for staff nurses in Queen Alexandra's Imperial Military Nursing Service which will be filled at once. The salary starts at £40 and rises by annual increments of £2 10s. to £45. Applications may be made in writing to the Secretary of the War Office, 68 Victoria Street, S.W., or personally to the Matron-in-Chief at the same address. The latter will see candidates on Tuesdays and Thursdays between the hours of 10 a.m. and 1 p.m.

### CRICKET MATCHES AND DISTRICT NURSING.

The cricket match organised by Mr. Thomas Hayward for the benefit of the Cambridge and District Nursing Institution came off on Saturday in the presence of some 7,000 spectators. Lord Dalmeny travelled specially from Scotland in order to take part in the contest, and ran up a score of sixty-nine. The amount realised was upwards of £61, a result which suggests that cricket matches on behalf of nursing associations might be got up with advantage elsewhere.

### HOME FOR INVALID FRENCH NURSES.

It is mentioned in the September number of the *Bulletin Professionel des Infirmières et Gardes-Malades* that on July 31 a county home was opened in the beautiful Chevreuse Valley for sick and overworked French nurses, where they can enjoy rest and repose and good air. The idea originated with M. Mesurier, to whom France owes so much of the progress made in nursing matters. Nothing is stated as to whether the institution is free, open to all nurses, or for any special class, nor the number to be accommodated; but evidently care and thought for the French nurses are steadily developing.

### THE DUCHESS OF NORFOLK'S ASSOCIATION.

Changes of an important character will be proposed at a meeting next month of the subscribers to the Duchess of Norfolk's Nursing Association. It is intended to suggest that in future the Association shall provide cottage-aid nurses for service in the town of Arundel and adjacent parishes; fully qualified nursing aid over the same area; and shall keep open-air emergency hospitals at which no serious case or accident will be refused. For all this work it is thought that one fund will suffice, the fund being controlled by a final committee composed of the Duke and Duchess of Norfolk and other members chiefly elected by the general body of subscribers. The two nursing committees, one dealing with the fully qualified nurses, and the other with the cottage-aid nurses, will be continued as heretofore. It is satisfactory to note that purely local efforts of this character, supported largely by private generosity, are now so often placed on a sound, administrative basis.

### ABERDEEN EYE INSTITUTION.

Last year Miss Boyd, the matron of the Bath Eye Infirmary, was chosen as matron of the Aberdeen Eye Institution, and took up her duties in December. She works single-handed, with a maid for domestic purposes, the number of beds being four, and of in-patients during the year about fifty. The first public meeting of the subscribers to the institution has just been held, and the report, which was read on the occasion, shows that it is doing excellent work. Professor Lang, who spoke at the meeting, mentioned that the attendances last year were close upon 11,000, that the new cases were 3,621, that 1,386 cases resulted from steel, stone, or other injuries, and that there were 736 cases of refractions; and said that this was a tale that commended itself to all. Allusion was also made in the report to Miss Boyd's excellent management, and it is evident that she has found a congenial sphere.

### SHORT ITEMS.

Mrs. Shaw has forwarded to the treasurer of St. Bartholomew's Hospital £1,000, half of which is for the general building and the other half for the new Nurses' Home fund.

### The Nursing Outlook.

"From magnanimity, all fears above;
From nobler recompense, above applause,
Which owes to man's short outlook all its charm."

### THE USE OF BATHS AND BATHING.

The enforcement of the doctrine of cleanliness in a modern hospital is of the first importance. This must be obvious when consideration is given to the enormous reduction in the mortality in hospitals, to the great diminution in the number of days each patient is now resident, and to the advantages which the whole community have experienced in consequence, both in matters of health and in the saving of money through the added earning power thus given to the artisan classes especially. Hospital committees sometimes display, however, a remarkable ignorance in the hasty decisions at which they arrive. No reasonable person would have thought it possible that a hospital committee, for instance, could have had any doubt as to the importance of providing ample baths and bathing accommodation for the nursing staff. Much less will it be credited that such a committee could be a party to the enforcement of a rule prohibiting any nurse from taking a bath without the special permission of the matron first obtained. Yet such things have happened and are happening to-day in some of the hospitals in this country.

Much wonder was expressed when the Raphael Nurses' Home, known as the "marble palace," was opened for the accommodation of the nursing staff of Guy's Hospital. This is a memorial home, and the whole of the cost was provided out of the private purse of the family who in this way desired to commemorate the memory of one of their dear ones. No doubt wealthy people may sometimes waste money in memorials, but in the circumstances we held the opinion that the Raphael Home was a worthy monument to the noble desire to commemorate good work well done by a departed friend. Much criticism was directed to the circumstance that the Raphael Home contains a large swimming bath where the nurses can learn to swim, and where they have the fullest opportunity to bathe in their off-duty time. The effect of this provision upon the health and efficiciency of the nursing staff of Guy's Hospital cannot be questioned, and we shall be surprised if the swimming bath does not take its place in process of time as a desirable addition to the equipment of the larger homes for nurses in great cities at any rate.

The conditions and circumstances under which a nurse has to work in a great hospital render it essential that she shall pay the closest attention to personal hygiene, and that in regard to her ablutions she shall take infinite pains to wash and be clean. Indeed one of the great advantages of modern nursing in hospitals has been the effect it has had in securing improved hygienic conditions and the enforcement of the closest attention to cleanliness in connection with everything that has to do with the wards and the patients. In such circumstances it will hardly be believed that the committee of a large hospital in the provinces, when seized with an economical fit, first decided to substitute for ordinary fresh butter a blend of English and Colonial butter at 1s. per lb. By such a step this committee invited discontent amongst the staff, for parsimony is never true economy where efficiency is the first consideration, as it must be in a great hospital.

They next directed their attention to the water supply, and apparently came to the conclusion that the nurses and servants had too nice a regard to personal cleanliness. The committee held that the nurses were using too much water and too frequent baths, and they consequently acceeded to the matron's application to have locks put on the bath-room doors, so that no one could have a bath without first obtaining the key from the matron. These proceedings caused great distress in the minds of the nursing staff, and they memorialised the medical board of the hospital. The medical board instituted inquiries, and found that the nurses desired to have a bath every day, but the authorities considered that one bath a week was ample. Hence the locked doors to enforce the latter point. In the result the medical board ordered the locks to be taken off the bath-room doors, and the nurses have since had reasonable opportunities for promoting personal cleanliness and comfort. The committee however still hold, we are informed, that a daily bath for a nurse is extravagant and wasteful, and they object to it because the consumption of water involved represents an average cost of some 30s. per week for the whole staff.

The facts we have recorded enforce the suggestion we made last week that it is of the first importance that steps should be taken to organise a course of lectures by some representative authority which will give those who aspire to be members of a hospital committee an opportunity of learning something about the subjects with which they will have to deal in such a capacity. When a committee has so little sense as to struggle with its nursing staff over an expenditure of 30s. per week, and to save this they sanction steps to prevent the nurses paying due regard to personal cleanliness, it is time for the medical staff to interfere, and for the governors immediately concerned to take steps to secure that the affairs of their institution shall, at the earliest possible moment, be placed in more competent hands.

### The Care and Nursing of the Insane.

By Percy J. Baily, M.B., C.M.Edin., Medical Superintendent of Hanwell Asylum.

### I.—ANATOMY AND PHYSIOLOGY.

(*Continued from page* 343.)

Now, certain portions of the grey matter of the convolutions exercise command over certain definite groups of muscles. What I mean is this, when we move our toes, the nervous impulses which result in such movement arise always from the nerve cells within a limited area of grey matter which is situated at the summit of the cerebral hemisphere, a little in front of the middle. Just outside this is a small area of cells which control the muscles which move the knee, and outside this again, those of the hip, and so on. These patches of grey matter, which, of course, cannot be distinguished from one another by any visible difference, are known as the motor areas.

Every movement we make, then, we make with our brains, the muscles being merely the mechanical means through which the brain acts, and if from any cause, as frequently happens in disease, the muscles are prevented from receiving their proper stimuli from the motor areas, they become paralysed and useless.

It is the same with sensation—we taste and feel, not with our tongues and skin, but through these with our brains. If, in the case of one who is sewing, the point of the needle should happen to enter the skin of the finger, the sewer at once becomes conscious of the fact because she feels the prick, in other words, she becomes conscious of a painful sensation. The point of the needle stimulates the end of a sensory nerve in the skin, this gives rise to a nervous impulse or message, which is conveyed along a sensory nerve-fibre into the spinal cord, and so through the white matter of the brain to the cells in the cerebral convolutions. When this impulse or message reaches the particular cell or group of cells whose business it is to receive and interpret messages from the point of the finger, and not till then, we become conscious of a painful sensation; if through disease these particular cells should be destroyed, or the nerve-fibre in any part of its course interrupted, all sensation in the finger would be lost and one might stick needles into it and feel no more pain than if one were sticking them into a pincushion, although the appearance of the skin might not be altered in the least degree.

The grey matter of one cerebral hemisphere controls the movements and receives the sensory impressions from the opposite side of the body. The reason of this is that the nerve-fibres in their passage through the spinal cord cross over from one side to the other, so that those which come from the nerve-cells of the left side of the brain pass to the muscles of the right side of the body, while those which come from the skin of the right side of the body convey sensory impressions to the cells of the left side of the brain.

2. *The Functions of the Cerebellum.*—This portion of the brain appears to be concerned chiefly in harmonising the various muscular movements and in balancing the body. A pigeon whose cerebellum has been removed, although still quite capable of voluntary movement, cannot effect any definite or regular purpose. Instead of flying it flutters, the movements of its wings being clumsy and irregular; there is, however, no paralysis or loss of intelligence.

3. *The Functions of the Pons and Medulla.*—The grey matter of the intermediate brain contains many nerve centres which control various more or less unconscious movements which are of vital importance. Such, for instance, are the movements of respiration, the movements of the heart and blood-vessels, the act of swallowing, etc.

4. *The Functions of the Spinal Cord.*—From what has been already said, it will be easily understood that the white matter in the spinal cord consists largely of nerve fibres on their way to and from the brain, so that it is obviously a great conducting medium. The cells of its grey matter carry on many functions, of which we need only mention two: (1) they exercise a great influence over the nutrition of the muscles, and (2) they are the centres for

*Reflex Action.*—A reflex action may be defined as an involuntary or unconscious movement which is dependent for its causation upon an afferent (sensory) stimulus. It will be better understood by an example. If, for instance, a finger is suddenly pricked with a needle, the hand is instantly withdrawn, or if a light be suddenly flashed before the eyes the eyelids are involuntarily closed, or when the feeding teat is placed between a baby's lips the baby immediately commences the movements of sucking. All these actions are performed without the intervention of the will and are examples of reflex action.

For the carrying-out of every reflex action, certain definite structures or tissues are required. These are (1) a sensory surface for the receipt of the stimulus. In the above instances this consists of the skin, the retina, the mucous membrane of the lips.

(2) An afferent nerve which conveys the stimulus from the sensory surface to

(3) one or more nerve cells (a nerve centre).

(4) An efferent nerve which conveys the (reflected) impulse back from the nerve centre, to

(5) one or more muscles.

Many of the ordinary actions of our daily life are carried out reflexly. A large number of them have for their object the preservation of life or the prevention of injury, or the removing of some irritating or deleterious object. As examples of the latter may be mentioned sneezing, coughing, and vomiting.

*Common Sensation.*—In its waking state the brain is continually receiving a set of stimuli or sensations through the skin. Such are the sense of touch, the sense of pain, of heat and cold. These are together spoken of as common sensation.

From the muscles, also, the brain receives afferent impulses whereby we are enabled to judge of the amount of contraction which takes place in them when we move, and also of their position. This is the muscular sense, and by it we can tell without using our sense of sight, whether our limbs are flexed or extended.

*The Special Senses* are sight, hearing, taste, and smell, and for their reception we are provided with special organs, which in the case of sight and hearing, at least, are of an extremely complicated kind; less so in that of taste and smell. Into the structure of these organs it is not necessary for us to enter.

It is through these various senses that we become cognisant of our surroundings, and, as we shall see in a later section, the proper development of the mind depends very largely upon them.

II. *The Sympathetic Nervous System.*—This consists of a double chain of nerve swellings or ganglia, as they are called, lying in front of the bodies of the vertebræ in the neck, thorax, and abdomen. Each ganglion is connected to the one above and below it and to its neighbour by nerve fibres. Connected with this chain are networks or plexuses of nerve fibres scattered among the thoracic and abdominal contents. The sympathetic system receives fibres from and is under the governing control of the cerebro-spinal system. It is concerned chiefly in the regulation of various vital processes of which we are ordinarily more or less unconscious, and over which we are unable to exercise any voluntary influence. It supplies the involuntary muscles of the heart, arteries, stomach, intestines, etc.

### The Nurses' Clinic.

### INJURIES OF THE UPPER LIMBS.

Injuries to the arms are common, and the same treatment may apply for bruises and cuts as for the lower limb. Cut tendons of the fingers and hand will need a surgeon's skill, but the nurse may be required to cleanse the wound and prepare surrounding skin for operation. Plenty of hot water and soap are the first and foremost agents; then a liberal application of carbolic, 1 in 80, or hyperchloride solution, 1 in 1,000. If there is much laceration and the wound is deep, the patient will probably be anæsthetised before all this is done. If the bleeding is not severe, and there is much danger from the nature of the accident of the presence of dirt and grit, a bath of some antiseptic lotion may be ordered for an hour or two previous to the sewing up of the wound.

A splint will nearly always be required for any cut or wound at all severe, so that movement will be impossible, and the rest so necessary to the prevention of inflammation may be secured. Dislocation of the thumb or fractures of the thumb or fingers will need the support of a splint, and the surgeon may order massage and movements to be done after a few days.

Fractures of the arm do not as a rule necessitate that the patient should remain in bed, so that the nurse's tender care will probably be required only so far as seeing that the splints keep in place and reporting any discomfort or swelling more than is usual.

Here again the nurse may learn a good deal about splints, for, as in the lower limb, the various fractures require various splints.

The "Colles'" fracture, commonly called "fractured wrist," is usually put up on a "Carr's" splint. The length varies with the length of the forearm, and the upper end of the splint is slanting; the fingers bend over, thus providing a little extension on the radial side.

For a fracture of the radius or ulna, or both bones, a straight splint is sometimes used, or an anterior angular splint, and the arm supported in a sling.

Fractures of the olecranon are often wired, this being a fairly certain method of providing the apposition necessary for good union. Fractures of the humerus are usually put up with the elbow flexed, so as to keep the muscles attached to the bone at rest. Anterior and posterior angular splints may be used, and the arm bandaged to the side or put in a sling.

In nursing patients suffering from injuries of the limbs the nurse will have plenty of practice in the art of bandaging, for it is indeed an art, and one which some nurses are apt to treat with indifference; but the excellent results, both from an appearance and a comfort point of view, will sufficiently reward the careful and painstaking nurse, and she will readily acquire the habit of good and rapid bandaging. It is necessary when practising bandaging to do so always with dressings or splints, otherwise when the actual time comes for a firm and good bandage over dressings the operator will be handicapped.

To know the pressure points for arresting hæmorrhage in the upper limb is very essential, and the careful and thorough nurse will see that she possesses the needful knowledge, so as to be ready for action when required.

### Incidents in a Nurse's Life.

### HOLIDAY DUTY.

I am not a lover of holidays, but a change of some sort is necessary, so as the least evil I decided on a "holiday duty." On my way to take up my temporary sphere of work I spent a few hours at the Cathedral city where I had to change, and after a quiet rest and a cup of tea, to my surprise I began to feel quite resigned to holiday making.

I had time to attend service in the Minster. How grand and solemn it was! and how I enjoyed it all! The peaceful evensong in the choir; the saintly Dean; the gay dresses of the ladies against the oaken background; the important verger as he marshalled the clergy to their places with his rod; the quiet saunter round afterwards with other holiday folk, followed by a walk through the steep narrow streets, with quaint low-windowed shops full of relics of the town, back to the station, and on to my destination through the beautiful flat country with its canals, fat cattle, and lovely golden cornfields.

The district nurse had already departed and I found I truly had a holiday duty—only one old lady whose digestive troubles had become endeared to her and proved a source of income to her from the Lady of the Manor. But there were midwifery cases expected. One was realised in the early hours of next morning. A quiet walk with my cycle over the fields towards one of those quaint little cottages dotted about the country, dressed in white coats and red hats, with a brown-faced husband (who was not in the least excited at the coming of No. 4) brought us to the family cabbages. Here the welcome sound of a baby voice told me that the little stranger had arrived. With the help of a pleasant old

INCIDENTS IN A NURSE'S LIFE—*continued.*

country woman my duties were soon over and I was cycling back through the country lanes just as the day was breaking, and I believe subsequently continued my dreams of the previous day just where they had been interrupted.

Then on Sunday the peaceful eight o'clock service in the tiny church, with the fresh country air and smell of sweet things coming in the door, and the eleven o'clock service in the larger building, with the silken drapery of the semi-invalids rustling in late to disturb our prayers, and who for their sins had great difficulty in finding seats. I could not help feeling amused at the conscious pride of the lady visitor in front who is the possessor of a pretty daughter over the lady who sits behind who comes alone. And our vicar, beloved of his flock, who does not aspire to preach, so talks gently to us for about fifteen minutes, with occasionally politely wrapped up thrusts at the visitors. And, oh! the pleasant rides down the nice straight lanes with the honeysuckle climbing over the blackberry bushes across the dyke just beyond one's reach; the purple heather; the rambles to the ruined abbey and castle; and the excursions to neighbouring villages which are all four miles away—sometimes "four gude moiles," but always the same number—I found all was delightful.

I had more patients after a time, but they all had holiday complaints which soon passed away; even the poor cancer woman was exceedingly comfortable, most cheerful, and doubting whether she had anything the matter. I fear that ere long the poor thing will change her opinion.

My holiday duty over, I fell to wondering if my village might not be a little dull after the country has put off its summer clothing, the visitors departed, and the villagers given up to gossip, gramophones and piano playing, and I gladly take comfort in the thought as I return that after all "there is really no work like one's own work."

### Some Impressions of my First Operation.

BY A MALE NURSE.

"Well Jenkins, how are you this morning? Do you feel fit?" "Oh yes, doctor, thank you. Do you think I am going to be all right, sir?" "Oh dear yes, of course you are."

The first speaker is the House Surgeon, and the other a young fellow, lying on a trolley waiting to be wheeled into the anæsthetic room, preparatory to undergoing trephining. It is nearly nine, and we are waiting for the appearance of Mr. B——, the surgeon, who is always punctual. Jenkins is quite cheerful, but anxious to get it all over. He is hemiplegic, which is considered to be due to a tumour of the brain, and which the surgeon hopes to be able to remove. At last footsteps are heard on the stairs, and as the surgeon goes to change his boots, etc., we wheel the trolley into the anæsthetic room, and prepare for the anæsthetist. Soon we are quite ready. Jenkins's bandages are taken off, so that the compress may be speedily removed in the theatre.

Dr. R—— comes in to administer. The mask is applied, and the patient is told to breathe quietly and naturally. Jenkins gradually sinks into unconsciousness, at the same time expressing his determination in song not to leave "my little wooden hut for you." The song soon dies away, and Jenkins sleeps, to wake no more till the operation is over, and he is safely in bed again in the ward.

Now the theatre doors are opened, and the trolley brought in close to the table. Ready hands assist to lift the patient on to the table, and everyone then takes their place. There is no confusion and very little talking. To the novice it is almost a weird spectacle. The most imposing person is the surgeon himself, in his long white mackintosh overalls, india-rubber gloves, white cap, and gauze veil over his face, with two holes cut in it, through which can be seen his keen eyes peering through gold-rimmed spectacles. Then his assistants round the table are all clothed in white overalls.

Hardly a sound is heard, except the stertorous breathing of the patient and the rattle of the tiny valve in the Vernon Harcourt inhaler. To cough is a crime, which merits instant dismissal from the theatre. No one speaks except the surgeon. Sister only beckons; she has no need to do more, as every one knows exactly what is expected of them. Now and again the surgeon's voice can be heard, "Sister, dry swab," "more lotion," "towels." Nurses are moving swiftly about with porringers and jugs, and filling irrigators with lotion.

Now the sun is getting up, and the temperature begins to rise, as no windows are open and no air can enter because of the deadly microbe. At last we hear the surgeon ask for sutures, and we know that the operation is almost finished. The wound is stitched, dressings applied, and Jenkins is lifted on to the trolley and taken back to bed.

### The Nurses of the Isle of Wight County Hospital.

INTERVIEW WITH THE MATRON. BY OUR COMMISSIONER.

Coming off the pier at Ryde, I noticed a small crowd upon the pavement, nearly at the bottom, and, drawing closer, found that the centre of attraction was a large dog carrying a collecting-box on his back. He was barking to attract attention, and as soon as one coin was added to his funds, he approached another passenger and again asked for more. Any pennies thrown to him he cleverly caught in his mouth and laid them down at his master's feet, who in turn slipped them into the box, which was labelled "For the Funds of the Royal Isle of Wight County Hospital, Ryde."

### How the Dog Helps the Hospital.

This incident I mentioned to the Matron, Miss E. C. Antram, when I saw her later in her sitting-room overlooking the pleasant lawn which fronts the hospital where, as I approached, I had noted some convalescent children enjoying themselves.

"Oh, yes," she replied, "the collecting dog, 'Nelson,' is a well-known feature of Ryde. He is owned by a shopkeeper, a former patient here, who, out of gratitude for what was done for him, has trained the animal to apply to the passers-by for money, and in the summer he often adds as much as 14s. or 15s. a week to our funds."

"I am afraid," continued the Matron, "that you have not come at the best time of the year to visit us, as, in consequence of building operations, we are keeping our numbers as low as possible. A new out-patients' department and a new isolation block, to hold two men and two women are in course of construction, and both are greatly needed."

"These are not the first additions since you came though, I believe?"

Recent Improvements.

"No. I have been here five and a half years, and before that I was six and a half years at Winchester, where I trained, ultimately becoming Sister. Just before I came here the children's ward, which is in memory of Queen Victoria, has been built, new sanitary blocks, with up-to-date bathrooms and lavatories, have been added both to the men's and the women's quarters, and a memorial hall has been erected to connect the children's ward with the main building. Before then it was quite distinct, and the nurses had to cross in the open in all weathers. The women who used to come in to clean the wards have been abolished, and wardmaids have been substituted all over the hospital."

"The three years' training was in force when you took up office?"

Previous Training Objected To.

"Yes. Probationers are not received younger than 23 years of age, and I much prefer that they should come straight to me without previous training of any kind. I find that those who have had experience in fever or children's work first have occasionally to unlearn, which is bad for them, bad for those over them, and for those in their charge. No premium is required, and no salary is given the first year; but they are provided with material for indoor uniform and laundry. Out-door uniform is optional, but, if they wish to wear it, they are required to wear a dark blue cloak and a dark blue bonnet, so that they may all be alike. The second year they receive £14, and the third year £18. At the end of that time, if they have successfully passed their examinations, they are given, with their certificate, a badge, which becomes their own property, and which they are required to wear at all times. It is made of silver, and has the letters of our Hospital, "R.I.W.C.H.," simply intertwined. If considered desirable at the end of the three years, the nurses sometimes remain on for six to twelve months to act as staff nurses at £25 a year. I am glad when I can to promote them higher still, as I prefer the sisters of the hospital to have been trained here."

The Convalescent Home.

"Of what number does you staff consist?"

"Of five sisters, one night, three day, an out-patient and casualty sister,—who has been here for 30 years,—and 11 probationers. Of these latter sometimes one or more rank as staff nurses, but that is just as it happens. We have also a lady dispenser, who has been here six years. The Matron of the Ryde Convalescent Home—which is connected with this hospital by a covered corridor—acts as my assistant, and takes my place here as Matron when I go for a holiday."

"Is the Convalescent Home for the benefit of those in the hospital?"

"Any patients here whom we think likely to benefit by a stay at the home may be sent there when they leave here; but otherwise it is open to those who need rest and change, the inmates—ten in number—coming from all parts. They have to obtain a subscriber's letter and contribute £1 1s. for the time of their stay—namely, 7s. a week."

Accommodation for the Nurses.

"You did not mention the sister's salaries?"

"They start at £28, rising to £30 the second year, £32 the third, and £36 the fourth. They have bedrooms to themselves, and some of the senior nurses have the same. But most of the probationers are obliged to share a room. When this is the case I try to place friends together, and to arrange that their off-duty time shall correspond. With regard to the first point, strangely enough, nearly all my nurses come from the North of England, so that they are not always strangers to each other. As to the second, on the monthly holiday the nurses are allowed to have breakfast in bed, and in the winter to have a fire all day. Their pleasure in these two luxuries is naturally enhanced if they have someone else with whom to share them. So, if possible, we arrange the monthly holiday of room-mates to coincide. Sometimes on these occasions leave is given for a tea party upstairs, which is an additional boon."

"What are the hours of duty?"

"Breakfast at 7 o'clock, in the wards by 7.30, dinner at 12.30, tea 4.30 and 5.30, and all day nurses off duty by supper time. As the doctors attend here a great deal in the afternoon, many coming over from the mainland, the nurses' off-duty time alternates between mornings 10 to 12.30 and afternoons 2.30 to 4.30. There is always three hours off on Sunday. The sisters have similar off-duty time, except that every fortnight they are free at two o'clock on Sundays."

Night Work and Operations.

"What about night work?"

"It depends principally upon the probationer herself how soon she begins night nursing. If she appears fit to do so she takes it up at three months, if not she waits till the end of the fourth or fifth month. There is one nurse to each floor, men, women, and children, besides the night sister. I am an advocate of a probationer becoming accustomed to night work fairly soon in her career. It seems undesirable that a nurse with perhaps twelve months' experience, who is fairly well up in many things, should be almost incapable of undertaking night work, as must occur if the probationers are kept entirely to day nursing for a long time."

"And operations?"

"We have a great deal of that work here, for, except the Hospital at Cowes, which is much smaller, we are the only operating hospital in the island. I am always present at the operations, but during her second year each nurse has charge of the theatre for three months at a time. In this way the nurse gains, to my mind, valuable experience, experience which it would be impossible to obtain were we to employ one nurse as theatre sister. A probationer who merely watches and assists the theatre sister learns only half as quickly as when she feels that she is the responsible person herself. During the winter months probationers have lectures given them by the house-surgeon."

Registration.

"One more question before, as you kindly suggest, I see over the hospital. Are you an advocate of registration?"

"No," she said laughingly, "I do not think I am, because I cannot see that any definite good will arise from it. Many of the best nurses are very bad at expressing themselves on paper; these nurses would be at a great disadvantage. Moreover, it would be impossible to provide against deterioration; once registered would be always registered. But it is not a subject I am very keen about, one way or the other."

A Harmony in Green and White.

Then the Matron conducted me to the children's ward, to reach which we passed through the new memorial hall, which has been erected by a lady in memory of her husband. It is a harmony in green and white, tiles and mosaic, with pretty glass windows, in which gold colour is introduced very effectively. The ward itself, which contains ten cots and one bassinette, is exceedingly pretty and very bright, with all modern improvements, and a large sun parlour, all glass, at the end for the children to play in during the winter. For really cold days there is a big stove as well, but it is not often necessary to use it. One cot has been endowed by the pennies collected by the Sunday-school child-

### THE NURSES OF THE ISLE OF WIGHT COUNTY HOSPITAL—*continued.*

ren of All Saints' Church, Ryde, and I noticed that all the cots let down on both sides to facilitate nursing.

Royal Visitors.

Before leaving the ward I was requested to write my name in the hospital book, where are the signatures of the late Queen Victoria, King Edward, Queen Alexandra, Princess Henry of Battenberg, and the present Queen of Spain. On my way back I observed the three outside iron staircases, which in case of fire can be lowered with one turn of a wheel, and visited the male and female wards.

Small Wards.

Altogether there is accommodation for 54 patients, but owing to the building being old, there are no very large wards, and the most roomy only contains seven beds; the smaller three or four. In respect to store-rooms the hospital is very well off, and I was shown a room kept expressly for splints, cradles, and crutches which a very large institution might be proud to possess. Another room is devoted to patients' clothes, a third to linen, and everywhere the things are neatly stowed away on shelves made of narrow pieces of wood with a good space between each to allow of a free current of air. The Matron is no believer in either close cupboards, solid shelves, or immovable fixtures. She showed me with pride the new marble-topped lockers in some of the wards, which are specially ventilated, and made with a loose shelf that can be taken out and scrubbed on both sides, and turned over for use so as to avoid warping. Later we peeped into another of the quaint little rooms in which the hospital abounds. This has been arranged for the use of the nurse on duty. It leads just off one of the wards, has a nice little table, with a bright cloth on, where letters can be written, splints padded, needlework done, whilst around hang the cups and saucers, etc., needed for breakfast and tea. It might be described as a "pantry-sitting-room."

As I passed out I felt that I had seen a hospital which posesses a pleasant atmosphere of home.

### A Nurse on Health.

The blind cannot lead the blind. The sick cannot tend the sick; a nurse must be healthy in body and mind to fulfil her vocation. Her personal atmosphere should brace and quieten; she must have the composure of reserve strength, and this is not possible if she is living up to the extreme limit of her powers. Some, if not many women, in every busy hospital do their duty in the wards listlessly and heavily, without any real pleasure in the work and without the smile and word for their patients which make all the difference to them; and this because of utter weariness of body and spirit, not because of lack of interest in either work or patients. "Health is contagious as well as disease." We have often heard one in weak health say, "I always feel better when you come in"; but "I didn't like to ask you, nurse, you were looking so tired," coming from a patient who has suffered for want of some attention—though it is little meant as such—should come as an insult, or, at least, as a rebuke, to the nurse. For she is there to be strength for her patient's weakness; to relieve, to lift burdens, not to obtrude her own.

Up to a certain point healthiness is ours to have or not as we will. The question is whether we think enough of what we owe to ourselves and our hospital in the matter. Every human being owes it to himself to keep as fit as possible. With lowered vitality comes invitation to disease, comes depression of spirits, comes, very easily, lowered moral tone. Life is difficult enough; we have no right to make it more so. And certainly a woman whose profession is that of nursing, an indispensable part of whose stock-in-trade is health, should not neglect the simple enough rules and precautions upon which her fitness for work may depend. That many of us do neglect these things there is no doubt. Tired when we come off duty, we take the path of least resistance, and lounge away the precious hour or two in our particularly small bedroom, when our whole being is calling aloud for oxygen, sunshine—sunshine at any price! On night duty we need more of bed than on day duty. The life is unnatural, the sleep less sound; we must have more rest. Instead of which some of us are frequently not more than five or six hours in bed, in preparation for our twelve hours' stretch of night-work. With regard to food—like other people—we eat too little of what is nourishing, too much of what is anything but nourishing, and most of us drink too much tea. It never seems to occur to us that this is, at least, hardly honourable, for, apart from what we owe ourselves, and apart from what we owe our patients, we owe much to our hospital. The attitude of mind is dishonourable, even where there is no actual evasion of rules. We have promised, in exchange for certain tuition and certain salary, to take on our shoulders a definite share of the work of the institution. We cannot carry out our part without our full health and strength. If this goes, we add to the work, instead of lessening it; and go it must if we deny ourselves the fresh air, sunshine, sleep, rest, and nourishment which we need.

Often illness is unavoidable, from infection, or accident, or breakdown, or where those in authority have imposed an unreasonable strain; but quite as often it is distinctly avoidable.

The carelessness is wilful, or it is only thoughtless, but a nurse has no right to be thoughtless. *Noblesse oblige.* I am thinking as I write of one bright, clever woman, who began her training in a big city hospital a few years ago. From the first, in spite of all argument, she treated her healthy body as if it were made of cast-iron, persistently took too little sleep, went out when she was far too fagged to walk, or stayed in when she needed fresh air badly, till she had the inevitable breakdown. It was slight at first, a warning; but things went on as before, and the second breakdown meant, after a long illness, a girl thrown on the world only half-trained in the profession which she really loved, and not fit to carry it on except in a very limited way. Ambitious for herself, she is left with no hope of reaching anything along the lines of hospital work, with no home, no income, no prospect even of having quite normal health again. A girl of great capability; who could most certainly, as far as it is possible to judge, have got through her training, with reasonable care of herself, and the proper husbanding of her strength.

I am not pleading for self-indulgence, for thinking constantly of our own bodily welfare, but for self-control. We can only save ourselves from revolt of our body by ruling it wisely. If we pay what is due when it is due there will be no arrears to make up with compound interest. This means self-denial, doing what is irksome, and doing it pretty constantly; but it is the old, inevitable exchange of losing our life to save it.

Hospital life is such a strenuous one; it is terribly engrossing, it sucks up every bit of us. But we have voluntarily sought it, and everything should yield, for the time being, to our fitness for living it well.

### The Nurses' Hostel.

### DISMISSAL OF THE LADY SUPERINTENDENT. STATEMENT BY MISS C. J. WOOD.

There is a strained situation at the Nurses' Hostel in Francis Street. A number of nurses who claim to be a considerable majority of the inmates are extremely distressed because Miss Hume, the new Lady Superintendent, who only entered upon her duties three weeks ago, has received an intimation that the Committee, who had met on Wednesday morning, wished her to leave in a week, and was informed that a cheque for a quarter's salary would be handed to her in lieu of notice. According to Miss Hume, she was astounded at the intimation, and at once demanded an explanation, which was refused to her; and all that she was subsequently told by the chairman was that she "had failed in tact in the management of the nurses." It may here be mentioned that previously to her appointment at the Nurses' Hostel, Miss Hume was successively out-patient sister and night superintendent at the Victoria Hospital for Children, Chelsea, assistant matron at the National Hospital for the Paralysed and Epileptic, sister at the Hospital for Sick Children, and lady superintendent at the Nursing Sisters' Institution in Devonshire Square for four years and a half, numbering 100 nurses. On her return from Ceylon, whither she went for a year on private business, she contemplated starting a home for nurses, but was asked to stay her hand, and waited accordingly for six months, when she was elected lady superintendent of the Nurses' Hostel, in the place of Miss C. J. Wood, who had retired. In the meantime she did superintendent's work in the south block whilst the permanent superintendent was away on her holiday.

The fact of Miss Hume's dismissal became, of course, the absorbing topic of conversation in the Hostel, and on Monday morning the nurses in the north block rose at breakfast and asked Miss Wood—who had returned temporarily to the Hostel—for an explanation. No answer was given, and then the nurses in the south block went over to Miss Wood to make the same request. She received one of them as a representative, and informed her "that the time was not ripe for a reason, but that in a most awkward position, she was trying her best to do fairly and justly." So far as the nurses are concerned, the action taken appears to be very much resented. It is claimed for Miss Hume that during the brief time she has been lady superintendent she has done everything she could to make the nurses comfortable, and, in particular, has effected a great improvement in the working of the telephone. There have, it is stated, been complaints for a long time that the nurses, though obliged to pay threepence for each message sent to them on the telephone, have not been allowed to reply to doctors by the same method; and after Miss Hume's appointment a petition, which was drawn up previously, was presented to her, signed by nearly 50 nurses, asking that they might be allowed to use the telephone themselves; that messages sent while they were out might be entered in the book; and that the rules of the Hostel might be printed in order that nurses might know what they were. This petition had already borne satisfactory fruit, and the nurses who signed it were looking forward to further alterations for the better in the general management, which, in several other respects, they assert, has for some time been defective. In a word, the nurses contend that they have been treated as school-girls or probationers, instead of being treated as responsible professional women earning their own living and paying their own way in the Hostel. They think that they are entitled to a full explanation of Miss Hume's dismissal, particularly as some of them are shareholders.

Miss Wood, in answer to inquiries, said that the question of Miss Hume's dismissal was considered by the Board for two hours. She herself had told Miss Hume—before the Board met—that she was not satisfied with the state of affairs, and thought that there was a lack of discipline and a disposition to stir up strife. She had not herself a word to say against the personal character or professional capacity of Miss Hume.

### The Accident to the Scotch Express.

### NURSING THE INJURED AT GRANTHAM HOSPITAL.

The matron of Grantham Hospital had just retired to rest for the night when the front door bell pealed. Hastily slipping on dressing-gown and slippers she ran down to see who had been admitted, and found two young men very dazed but apparently not much hurt, who explained that there had been an accident to the Scotch express just outside the station, and that the injured were being conveyed to the hospital. It subsequently transpired that a message to that effect had been sent by one of the doctors, but had failed to arrive. Quickly dressing, assisted by the day sister and a nurse who were called, she got everything in readiness to receive the first of the seriously injured. In the meantime the night sister and her probationer had got the two men to bed. The only way to make sufficient room was to rouse two convalescent men and to remove two youths to small beds in the children's ward. The male ward contains ten beds, seven of which were occupied at the time of the accident. All the doctors were at the scene of the disaster, the surgeon for the week coming with the first patient, who was quickly followed by two others. Altogether seven men, two women, and one child were admitted. There were only two empty beds in the female ward, but a third was quickly made available by sending a convalescent girl into the children's ward—which had to be somewhat overcrowded, for several cots contained two children—the two convalescent men being accommodated on couches. A sister of one of the doctors who is a nurse came with her brother and remained all night, being of the greatest assistance to the nursing staff. She has also attended each day through the week and helped. As the patients were admitted they were each quickly put to bed, surrounded by hot bottles, and covered up with plenty of blankets. All the beds are provided with full-length mackintoshes under the bottom sheets, so that none of the mattresses were soiled, in spite of the profuse hæmorrhage from some of the wounds. As soon as the last patient was in bed all clothes were removed, being kept separate by putting them under each bed, in order that there might be no difficulty in identifying the patients if necessary. When all had been undressed the doctors—there is no house surgeon attached to the hospital—thoroughly overhauled each patient, suturing all wounds and setting fractures. One was a fracture of the ulna, another a compound fracture of tibia and fibula, and a third a comminuted facture of tibia and fibula. All the patients had more or less severe scalp wounds, one having a fractured base and another being badly concussed. Most of the injured were able to have a preliminary wash, but it was not until two or three days later that they were made really clean. Various hypodermics were given and also hot and stimulating drinks, each patient being made as warm and comfortable as possible. The staff all worked steadily from 12 until 3 a.m. After this the clothes received attention, being thoroughly searched for marks of identification, money, etc., tied up in bundles, and labelled; while all valuables were done up separately and given into the matron's care. In this task the nurses were helped by the doctors and two clergymen of the town, who had assisted to make a list of the patients' names, addresses, and injuries. By 5.30 A.M. the matron and nurses retired to rest for an hour, and by 7.30 were ready, after a cold bath, to begin the day's work.

### Everybody's Opinion.

### AN INDICTMENT OF THE MIDWIVES ACT.

"Nurse G.," Oxton, Birkenhead, writes: Apropos of letter of inspector of midwives, I should be glad if someone could give me advice about beginning. I passed the August examination and spent most of my money in doing so. But the certificate of the Central Midwives Board does not seem much good without trained medical and surgical nursing attached to it, and it does not seem fair to advertise training schools and insist on so many rules if one cannot make a living of it. As a working woman I am willing and anxious to help my fellow-creatures at such a critical time as child-birth, and I am convinced that the rules of the Central Midwives Board make for good methods, having seen the effect during district training. But where to begin and how to make a living of it is another side to the question.

### MASQUERADING IN NURSES' UNIFORM.

The Matron of the Cottage Hospital, Porth, Glam., writes:—For weeks past four women, attired in nurses' uniform, have been scouring this district calling at the houses with tablets of "soap," and representing that the profits were for Dr. Barnardo's Homes. More recently still "pills" and "tea" have been similarly hawked about. A paragraph in a local paper shows that the same thing is being done in other districts. The other day my nurses were taking a walk. One of them—a locum for holiday work—had been purchasing some pictorial post-cards. As she passed a group of children one boy said, "Them nurses are selling post-cards now." If women are allowed to masquerade as nurses, surely there is no reason why clerical garb, postmen's, policemen's, and railway porters' uniforms should not be similarly affected. Personally we very much resent the indignity dealt to the nursing profession, and think that some strong measure should be adopted to put a stop to the practice. If the uniform were registered our association would be protected by law, and nursemaids, pedlars, etc., could not don the uniform unless properly trained.

### SOME ESSENTIALS OF SOUND ADMINISTRATION.

"A Matron of some Years' Experience" writes: The article on this subject in the issue of September 22 has many elements of truth, but it scarcely recognises the difficulties which obstruct the smaller schools from furnishing the full training necessary for a sister's responsibilities. Hospitals between 70 and 120 beds, more particularly those placed in quiet towns without manufactures, do not deal with sufficient cases to make really efficient sisters. The infrequency of acute cases, medical and surgical, the limitations of a small trained staff for the absence of the experienced staff nurse which forms so valuable an asset in our large training schools, but for whom no place can profitably be found in hospitals where, provided the training is for three years, the head nurse—by courtesy called sister—is usually equal to the requirements, and last, but not least, the type and age of the majority of applicants for the smaller hospitals. (It must be remembered how large and varied a selection is possible in our fine training schools with medical schools attached.) The article says: "If the training is such that the best and most capable nurses are not produced the whole establishment is a mistake." This is a sweeping statement, for most excellent nurses are trained in these country hospitals not always suitable for superintendence, but very valuable for the needs that do not always appeal to the nurses of our big schools. The private house, the district, the nurse for fever hospitals, and workhouse infirmary work. How many a devoted and tender nurse, who probably would have been a failure elsewhere, has not our country hospitals sent forth? The large well-organised hospitals have always more nurses than they can find head posts for. These nurses bring with them valuable lessons. They are more able to grasp emergencies, to deal with severe cases, and are better for discipline and teaching than the probationer, who, although possessed of her three years' certificate, has had but little scope for superintendence, and whose authority might be somewhat crippled by the too close intimacy and association which obtains in small hospitals. There is no reason why the introduction of a fresh element should always preclude the promotion of a capable nurse who has had some experience of *pro tem.* sisters' duties; and in hospitals where the sisters number more than six it may be possible to promote from the ranks, but even then she is the better for other and larger work first. It would be delightful to work a small hospital if that small hospital could provide the ideal.

### Appointments.

Carnarvonshire and Anglesey Infirmary.—Miss Alice Cunliffe has been appointed charge nurse. She was trained at the Royal Albert Edward Infirmary, Wigan, and has since been on the staff of the Oldham Nursing Association.

East Sussex Hospital, Hastings.—Miss Frances E. Smith has been appointed staff nurse. She was trained at the Middlesex Hospital, London, and has done holiday duty at Hove Hospital.

Open-Air Sanatorium, Wokingham.—Miss Ida Shuttleworth has been appointed staff nurse. She was trained at the Royal Hants Hospital, Winchester.

Sheffield Royal Hospital.—Miss A. M. Macer and Miss E. G. Pilkington have been appointed sisters, the former as sister of the out-patients' department and the latter as sister of the female surgical ward. Both were trained at the Sheffield Royal Hospital and have been staff nurses in the same institution.

Victoria Nursing Home, Shanghai, China.—Miss Evelyn Lea has been appointed matron. She was trained at the General Hospital, Birmingham, where she was afterwards sister of a women's ward. She has since been sister at the Women's Hospital, Euston Road, London. She had fever experience in the hospitals under the Metropolitan Asylums Board, midwifery training at Brighton, and she has a good knowledge of dispensing.

Walsall District Hospital.—Miss Grace Sutherland Smith has been appointed staff nurse. She was trained at the General Infirmary, Chester, and has since been at the Eye Hospital, Birmingham.

### Novelties for Nurses.

(By our Shopping Correspondent.)

### A BEAUTIFUL WOOLLEN FABRIC.

During the last few years a number of beautiful materials made of wool have displaced the old-fashioned flannel which was the sole material in use for underwear, whilst cashmeres and merinos ruled for costumes. One of the most excellent products of present times is Orlwoola, a delightful fabric, which is soft, firm, and fine. It is suitable for a number of uses—for night wear, under garments, costumes, and blouses. The designs are in great variety and colouring, and self-colours can also be had. Any nurse requiring woollen materials for the winter will be rewarded if she secure a collection of patterns from her draper.

### A Book and its Story.

RELIGION IN FICTION.*

The sudden passing away recently, under pathetic circumstances, of Mrs. Craigie (John Oliver Hobbes) gives a pathetic interest to "The Dream and the Business"—the last published novel by that gifted author. In a quotation taken from Ecclesiastes, "For a dream cometh through the multitude of business," she found a suggestion for the title of her last novel. The religious element has in this, as in other more recent works by this brilliant writer, a powerful influence on the development of the more interesting characters. It is well known that Mrs. Craigie's own religious experiences extended from Nonconformity to Roman Catholicism. "The Dream and the Business" the working of the two extremes is seen in Dr. Firmalden, a Bayswater Congregational minister, and Lord and Lady Marlesford. Lady Marlesford belongs to an old Roman Catholic family, her husband is the son of the first baron, who made a huge fortune by brewing and was a Protestant. Lord Marlesford at forty decided to choose a young aristocratic, beautiful, and inexperienced wife. He met Tessa Navenby at her first ball, proposed to her after a short acquaintance, and "two months later he was received into the Roman Church, and his marriage with Miss Navenby at the Brompton Oratory was the sensation of an Easter week in London." Between the proposal and the marriage an interval elapsed spent by Tessa Navenby in a convent, where she offered prayers for Lord Marlesford's conversion. He on his part had travelled. Finding many places tedious he directed his steps to Italy, with the result that at the first place he arrived at—Venice—he found that; "It seemed almost vulgar to be a Protestant; he hurried on to Florence. To be a Protestant in Florence is to be a tourist at best! He went to Rome. To be a Protestant in Rome was to be uncivilised, illiterate, and a shade ridiculous—so it struck him." His conversion was now only a matter of weeks. Outwardly, to the world, his marriage was a perfectly successful venture. But the position of the husband and wife was one which the author describes with the adroitness of which she is a mistress. They have been married three years, and "Lord Marlesford was not ambitious. He had for books and pictures a great taste, which, while it did not amount to an absorbing love, filled his time most agreeably and gave his mind its purest delight. As a county nobleman he did his share of hunting, horse-racing, stock-farming, and politics, and, being intelligent, he carried out in a satisfactory manner whatever he undertook, whether under the guise of duty or recreation. But enthusiasm he could not give to the national sports or to public affairs. He was, in his heart, a sentimentalist. Few guessed this; he was unconscious of it himself; his wife never knew it. At moments he suffered from a kind of melancholy which he was too proud to acknowledge and too sensitive to question. If a doctor of souls had told him that he was secretly longing to be better understood he would have thought him a prig or impertinent, or both. Nevertheless the doctor of souls would have been right. . . Handsome, amiable, tall, well built, and rich, he was rightly considered an exceptionally charming man, while Tessa was rightly considered the very luckiest of women. His character belonged to the virile type, but he was kind rather than passionate, reasonable rather than zealous, and keener to appreciate humour than the tragedy of events. His sense of humour was even extraordinary, inasmuch as he was also full of sentimental ideas and fancies, which, while they remained nearly always unuttered, ruled his conduct. Of all his sentiments the one he entertained for his wife was the most peculiar. He loved her, but they had not one feeling in common. They were close friends and talked together for hours at a time, yet each was a perplexing mystery to the other." Lady Marlesford's difficulty lay in the fact of realising that, in spite of his affection, Lord Marlesford did not understand her. Perhaps he idealised her character beyond its deserts. And she on her side seemed to have had little sympathy for the real Lord Marlesford, for her tastes were opposed to most things which delighted him. "She cared nothing about literature or art, except as manifestations of the world-spirit. She read books for their humanity and general information; she admired works of art for their decorative beauty. Music she disliked. She wanted to move on a larger scale and take part in some heroic drama. Her gifts were for a political career, and she ought to have married a Cabinet Minister or the editor of a powerful newspaper, or the leader of some revolutionary party. . . . She wanted power, and in order to obtain it she was perfectly willing to endure persecution, hardships, poignant disillusions, and ultimate ingratitude. But to live as Basil lives! one might as well be a squirrel in a cage!" This is the state of affairs when, through the chance meeting with Jim Firmalden, the Minister's son, who is travelling with his uncle, Sir Charles Banish, a Judge of the Queen's Bench, Lord and Lady Marlesford are brought into touch with the minister's family. Jim falls a victim to the charm of Lady Marlesford, with the result that a platonic friendship springs up between them. Lady Marlesford, interested in Firmalden's career, inquires, early in their acquaintance, what he intends to become. He replies, "A Nonconformist minister." "A Nonconformist! But why not a Catholic? I should like you to be a cardinal some day. I have been thinking—ever since you began to talk—that you were a man for Rome." "I could no more be a Catholic than you could be a Parsee," said Jim; "It is a question of temperament rather than dogma. If this truth were realised, there would be far less bitterness among religious people." "But surely," said Jim, "you are not a Papist?" "Didn't you know that?" replied Tessa. It was as though she had declared herself a professing witch. Each by a glance tried to pierce the real nature of the other. . . . Thus the friendship which had begun with a rapturous recognition of identical tastes assumed the colour of hostility. Her efforts on his behalf at conversion are not successful. But Lord Marlesford forms an attachment to Sophy, Jim Firmalden's sister, Lady Marlesford dies after the birth of a child. Her husband survives her and marries Sophy Firmalden, who becomes a Roman Catholic, after failing to reconvert Lord Marlesford to Protestantism. The impression left after reading "The Dream and the Business" is that of a series of studies in which one or both parties of ill-assorted couples at cross-purposes have either had a *grande passion* previously, or are seeking for one outside the limits of their present environment. Lessard, the handsome, wholly selfish, professional singer, who goes far towards breaking Sophy Firmalden's heart and rendering the lives of other women miserable, is an active factor for harm, in spite of his melting voice and irrestible manner. "The Dream and the Business" is a book that will produce pleasure or irritation in the reader's mind, in proportion to the sympathy, or lack of it, that is brought to bear on its contents. That it is clever, epigrammatic, and in parts witty, goes without saying; but that it has wide spaces that are tedious and dull even, and in strong contrast to the more brilliant passages which abound, is also true.

* "The Dream and the Business." By John Oliver Hobbes. Fisher Unwin. 6*s*.

### Notes and Queries.

**REGULATIONS.**

**The Editor is always willing to answer in this column, without any fee, all reasonable questions, as soon as possible.**

**But the following rules must be carefully observed.**

**1. Every communication must be accompanied by the name and address of the writer.**

**2. The question must always bear upon nursing, directly or indirectly.**

**If an answer is required by letter a fee of half-a-crown must be enclosed with the note containing the inquiry.**

*Sanitary Inspectorship.*

(295) I am told that I shall have little chance of obtaining a sanitary inspectorship unless I hold the Central Midwives Board certificate. Is this so?—*Queryist.*

If so it is quite a new rule. Write and ask the Secretary of the Sanitary Institute, Margaret Street, W.

*Nursing in Canada.*

(296) Is there any opening for English private nurses in Canada?—*Brighton.*

Not unless you have a friend in the medical profession and are acquainted with residents. There are very excellent training schools in Canada, where a large number of nurses are trained.

(297) Will you tell me if you think it likely that I and a friend can secure free passages to Canada by giving our services on the voyage? We want to nurse there, but as I have not a three years' certificate I should not mind going as a probationer.—*Anxious.*

You can advertise in the *Morning Post* and other journals offering your services, but we are sorry we cannot give you much hope of success either in this direction or in securing posts in the Dominion. Canada is well supplied with nurses. You can advertise in a Canadian nursing or other paper.

*Warts.*

(298) Can you tell me how to remove a seed wart from my finger? I have tried nitric acid, nitrate of silver, etc.—*Dublin.*

Try glacial acetic acid applied with the point of a sharpened match. But first shave off the hard tissue, which you probably did not do when you tried the other remedies. Be careful not to set up inflammation.

*Nursing Abroad.*

(299) Where should I apply for an appointment abroad? When does the season begin?—*Worthing.*

You do not say the locality abroad you desire. Get "How to Become a Nurse," The Scientific Press, 28 Southampton Street, Strand, price 2s. 4d. post free. You will find a list there of British nursing institutions abroad.

*Phthisis.*

(300) Can you tell me of a sanatorium or home where a case of advanced phthisis could be received for a fortnight? Fifteen shillings a week can be paid.—*Sister.*

Write to the Sister-in-Charge, St. Peter's Home, Mortimer Road, Kilburn, and ask if there is a vacancy at their Cheddar Home for Consumptives; to St. Barnabas Home, Brockett Hall, Torquay; or to St. Catharine Home, Grove Road, Ventnor.

*The C.M.B. Certificate.*

(301) In order to obtain the C.M.B. certificate must I undergo an examination, or can I be registered without? I was trained in midwifery at the East End Mothers' Home and hold that certificate.—*Mykes.*

(302) I should like to gain the certificate of the Central Midwives Board, but I am not sure I could gain it without going into hospital again; but I fear I cannot spare the time. I have had training in monthly nursing, and have had a good many cases. Can instruction be had by post?—*Leeds Luton.*

In order to practise as a midwife you must hold the certificate of the Central Midwives Board, and to gain this it is necessary to train at one of the institutions selected by the Board as training schools, or privately. You can find all information in detail in "How to Become a Midwife," The Scientific Press, 28 Southampton Street, Strand, 1s. 1d. post free.

**Handbooks for Nurses.**

| | Post Free. |
|---|---|
| "How to Become a Nurse: How and Where to Train." | 2s. 4d. |
| "Nursing: its Theory and Practice." (Lewis.) ... | 3s. 6d. |
| "Nurses' Pronouncing Dictionary of Medical Terms." | 2s. 6d. |
| "Complete Handbook of Midwifery." (Watson.) ... | 6s. 4d. |
| "Preparation for Operation in Private Houses." ... | 0s. 6d. |

Of all booksellers or of The Scientific Press, Limited, 28 & 29 Southampton Street, Strand, London, W.C.

### For Reading to the Sick.

SYMPATHY.

Brother, we are surely bound
On the same journey—and our eyes alike
Turn up and onward : wherefore, now thou risest,—
Lean on mine arm, and let us for a space
Pursue the path together! Ah, 'tis much
In this so weary pilgrimage, to meet
A royal face like thine : to touch the hand
Of such a soul-fellow; to feel the want,
The upward-crying hunger, the desire,
The common hope and pathos!

*Buchanan.*

When our Redeemer bore our griefs and carried our sorrows, He was travelling the road terminated by the Cross of Calvary. This thought must be our support and encouragement. The self-sacrifice which sympathy demands none knows as He knows, and when, having shared the sorrows of our fellow-creatures, we look for sympathy ourselves, none can be so able, or so willing to give it. All the tenderness, gentleness, and full comprehension of trial, which were His when He sojourned upon earth, are His still. He lives now, as He did then, in the life of those He loves. Blessed, indeed, shall we be when we can once realise this truth. No loneliness, no dreariness, for He is close beside us; no bitterness, for He understands what we cannot bring ourselves to utter; no weakness, for His loving eye is upon us, and in His glance is strength; no fear, for there can be no morrow of trial apart from Him. It is the lesson of years, slowly learnt, often forgotten, but it gives peace long before it is acquired perfectly. The moment when first in sadness of heart, we kneel in the solitude of our own chamber, unable, it may be, to express our grief in words, but only feeling the comfort of His presence, is a moment which brings a blessing to our whole lives. When we do not need prayer, when we do not want to ask help, when we only know that He understands. There lies the very germ of love—true, pure, deep, untold, unchangeable love, which will uphold us in death, love which will be our joy in eternity. If we, like Christ, would truly and rightly sympathise; if we would in our degree bear the griefs and carry the sorrows of our fellow-creatures, we must view those sorrows as Christ viewed them, and soothe them in His spirit. Let us remember that such sympathy is pain. It is not true sympathy unless it is pain. When we feel with and for another, we must in a measure suffer. Distant, indeed, infinitely distant, must be human sympathy compared with His, yet is it the same kind? God grant us each in our measure to strive after and attain it.—*E. M. Sewell.*

It came in gentle, loving deeds,
It came a flower 'mong bed of weeds,
And stood beside my fainting heart,
And showed we love in life had part.
In sympathy she smoothed my brow,
And ne'er will I forget, I trow,
This human flower that came alone
To cheer my way—a wand'rer—home.

